# Construction and validation of a predictive model for the risk of osteoporosis in patients with chronic kidney disease based on NHANES data

**DOI:** 10.1371/journal.pone.0316494

**Published:** 2025-02-06

**Authors:** Chunjie She, Hefeng Liu

**Affiliations:** Department of Orthopaedics, Chaohu Hospital affiliated to Anhui Medical University, Chaohu, Anhui, China; Sichuan University, CHINA

## Abstract

**Background:**

Chronic kidney disease (CKD) patients tend to exhibit a heightened susceptibility to osteoporosis owing to abnormalities in mineral and bone metabolism. The objective of this study was to develop and validate a nomogram for the prediction of osteoporosis risk in patients with CKD.

**Methods:**

1498 patients diagnosed with CKD were enrolled from the National Health and Nutrition Examination Survey (NHANES) data spanning 2005–2010, 2013–2014, and 2017–2018. The dataset was randomly divided into a training set and a validation set in a ratio of 7:3. Utilizing the least absolute shrinkage and selection operator (LASSO) regression technique for predictor identification, followed by employing multivariate logistic regression based on the selected predictors to construct a nomogram. The performance of the prediction model was assessed using various metrics, including the area under the receiver operating characteristic curve (AUC), calibration curve, the Hosmer-Lemeshow test, and decision curve analysis (DCA).

**Results:**

The construction of the nomogram was based on five predictors, namely age, height, weight, alkaline phosphatase (ALP), and history of fracture. The AUC of 0.8511 in the training set and 0.8184 in the validation set demonstrates robust discriminability. Furthermore, the excellent calibration and clinical applicability of the model have been thoroughly validated.

**Conclusions:**

Our study suggests a nomogram, providing nephrologists with a convenient and effective tool for identifying individuals at high risk of osteoporosis and avoiding adverse outcomes related to CKD.

## Introduction

CKD refers to the persistent impairment or decline in renal function caused by various factors for a duration of 3 months or longer [1]. This global health issue affects approximately 15-20% of the adult population worldwide and has seen a significant surge in prevalence in recent years [2]. Projections indicate that it is poised to ascend as the fifth leading cause of premature mortality on a global scale by 2040 [3]. Patients with CKD are frequently accompanied by a variety of chronic complications. The term Chronic Kidney Disease-Mineral Bone Disorder (CKD-MBD) is employed to describe the constellation of biochemical, skeletal, and extra-skeletal calcification abnormalities that manifest in individuals with CKD [4]. Osteoporosis is one of the skeletal manifestations observed in CKD-MBD, characterized by reduced bone mass and deterioration of the microstructure of bone tissue, leading to decreased bone strength and increased susceptibility to fractures [5–7]. Numerous prior studies have established a significant prevalence of osteoporosis and fractures among CKD individuals. According to the analysis of NHANES III data, the percentages of mild-to-moderate renal failure (GFR 35-60ml/min) was found to be 33.5% in women and 16.4% in men within the osteopenia group, while these percentages increased to 61.3% in women and 46.5% in men within the osteoporosis group. When considering a more severe degree of renal insufficiency (GFR <35 ml/minute) in the prevalence estimates, the result attained greater statistical significance, 85% in women and 57% in men [8]. When it comes to fracture, the incidence of hip fracture was 2.6 times higher in the group with a GFR <60 ml/min compared to the group with a GFR >60 ml/min [9]. As renal function deteriorates and progresses to end-stage renal disease (ESRD), patients with hip fractures may face a risk that is 4.1 to 17.4 times higher than that of the general population [10–13]. The presence of osteoporosis and fracture further contributes to the financial burden and mortality rates of CKD patients. The statistics indicate that the healthcare costs for patients with CKD after fracture exceed $60 billion. The in-hospital mortality rate of CKD patients with hip fracture was 3.7%, considerably higher than patients with normal renal function (1.6%) [14, 15]. Early diagnosis and prevention of osteoporosis are imperative in the management of CKD. However, there is currently a dearth of predictive models available for evaluating the risk of osteoporosis and bone fractures in individuals with CKD. The aim of our study was to develop a nomogram using the NHANES for predicting the probability of osteoporosis in patients with CKD, enabling clinicians to screen high-risk patients through easily accessible physical examination data and interviews, thereby ensuring prompt implementation of subsequent specialized examination and treatment.

## Methods

### Data source and participants

All data in this study were publicly accessible in the NHANES, which is a program of studies designed to assess the health and nutritional status of the United States population. The survey examines a nationally representative sample of about 5,000 persons each year through interviews, physical examinations, and laboratory tests. The collected data, after undergoing anonymization and coding, were released to the public. The study included CKD patients with complete findings of dual-energy X-ray absorptiometry(DXA). As NHANES does not provide a definitive diagnosis of CKD or an estimated glomerular filtration rate (eGFR) measurement, we utilized the Chronic Kidney Disease Epidemiology Collaboration (CKD-EPI) equation to calculate the eGFR [16]. In accordance with the Kidney Disease: Improving Global Outcomes (KDIGO) 2024 clinical practice guidelines, we defined CKD as an eGFR below 60 mL per minute (1.73 m^2^) or a urinary albumin-to-creatinine ratio (ACR) exceeding 30 mg per gram [1].

### Ethics statement

The NHANES has received approval from the ethics review board of the National Center for Health Statistics and obtained written informed consent from all participants enrolled in the research.

### Measurements and definition of osteoporosis

The osteoporosis cohort was identified based on the measurement of bone mineral density (BMD) at the femur and spine using DXA. Patients were excluded from the DXA examination for the following reasons: (Ⅰ)Pregnancy. (Ⅱ)Self-reported history of radiographic contrast material, such as dyes or barium, in the past 7 days. (Ⅲ)Measured weight over 450 pounds. (Ⅳ)They had fractured both hips, had replacements of both hips or had pins in both hips. (Ⅴ)The spine scan if they reported a Harrington Rod in the spine for scoliosis. (Ⅵ)Examination completed but invalid data. On the basis of diagnosis criteria established by the World Health Organization, osteoporosis can be defined as BMD values falling below −2.5 standard deviations from the reference group of young adults [17]. In our study, the evaluated regions were the femoral neck BMD and L1-L4 lumbar spine BMD, while the mean BMD of non-Hispanic white females aged 20–29 years was used as a reference [18]. Osteoporosis can occur at the femur neck, lumbar spine, or both.

### Predictor variables

We screened potential predictors from the NHANES database that may exhibit associations with the progression of osteoporosis in patients diagnosed with CKD. The selected variables, included sex, age, race, standing height, weight, BMI, standard biochemistry profile, renal function, vitamin D, HDL, smoking status, drinking status, hypertension, diabetes, history of fractures, and history of glucocorticoid use. Among the above variables, sex, age, race, smoking status, drinking status, hypertension, history of fractures, and history of Glucocorticoid use were obtained through a self-report questionnaire. Based on the number of cigarettes consumed and current smoking status, the classification of smoking status was as follows: no smoking (lifetime consumption < 100 cigarettes), quit smoking (lifetime consumption > 100 cigarettes but currently denying smoking), and current smoking (lifetime consumption > 100 cigarettes and admitting current smoking). Drinking status was categorized into three groups by drinking frequency: no drinking(Never in the last 12 months), low drinking (1 to 36 times in the last 12 months), and heavy drinking (>36 times in the last 12 months). Hypertension, history of fractures, and history of glucocorticoid use were determined by asking participants the following questions.“Have you ever been told by a doctor or other health professional that you had hypertension, also called high blood pressure?”, “Has a doctor ever told you that you had broken or fractured your hip/wrist/spine/any other?” and “Have you ever taken any prednisone or cortisone pills nearly every day for a month or longer?”.The response can be either Yes or No. The remaining variables, including standing height, weight, BMI, standard biochemistry profile, renal function, vitamin D, HDL, and diabetes were assessed through laboratory or physical examination. Diabetes was defined as glycohemoglobin level≥6.5%.

### Statistical analysis

**Fig 1:**
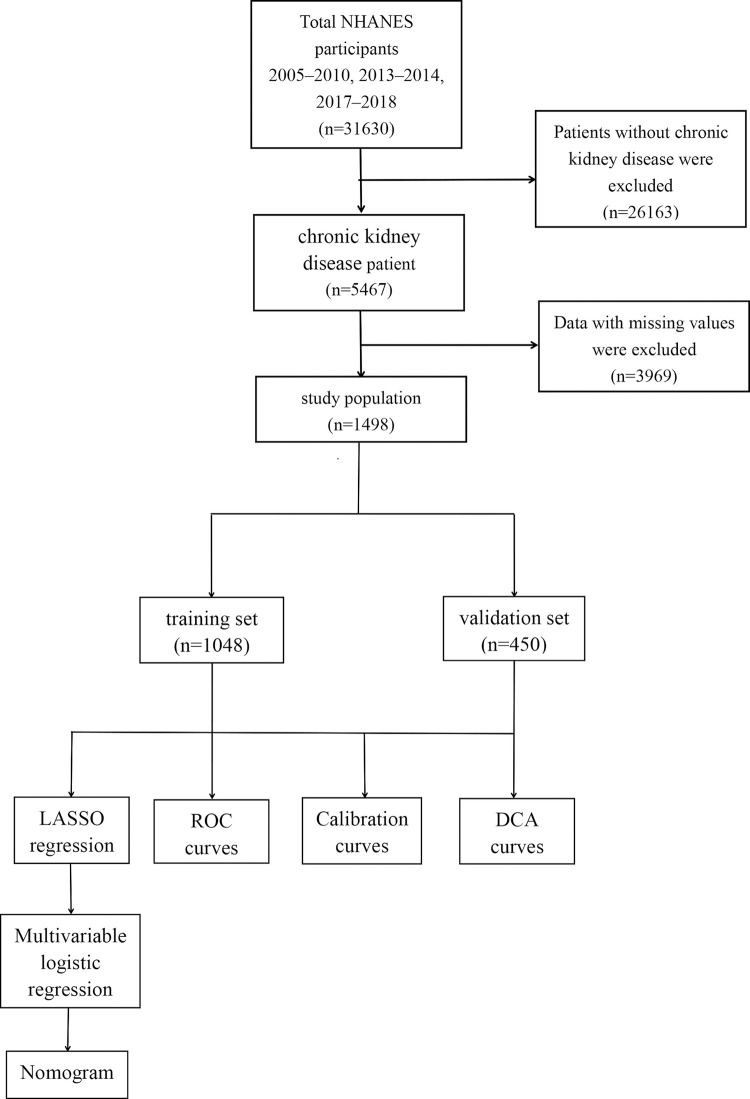
The flow chart of study population screening and statistical analysis. LASSO, least absolute shrinkage and selection operator. ROC, the receiver operating characteristic. DCA, decision curve analysis.

Statistical analysis was conducted by using R software (version 4.3.2). Continuous data and categorical data were expressed as the median (interquartile) and the number (proportion), respectively. Group comparisons were performed using a Student’s t-test, Mann-Whitney U test, and Chi-square test, for normal, skews, and categorical data. The dataset was randomly divided into a training set and a validation set at a ratio of 7:3.The optimal predictors were identified using the LASSO regression technique. Subsequently, multivariate logistic regression analysis was performed on the LASSO-selected predictors to confirm their significance and construct the nomogram. The AUC was employed to quantify the discriminative ability of the nomogram. The calibration curve and Hosmer-Lemeshow test were utilized to assess the agreement between actual outcomes and predicted probabilities. Additionally, the clinical utility of the model was evaluated through DCA. The process of study population screening and statistical analysis is illustrated in Fig 1. P < 0.05 was considered statistically significant.

## Results

A total of 1498 participants were enrolled in this study. Table 1 presents the characteristics of the CKD patients, divided into non-osteoporosis and osteoporosis groups. The overall prevalence of osteoporosis among patients with CKD was found to be 16.89% (n = 253). The comparison of patient characteristics between the training cohort and test cohort is provided in Table 2, with 1048 patients in the training set and 450 in the validation. Based on the statistical analysis results, no significant differences in baseline characteristics were observed between the training and validation sets.

**Table 1:** The characteristics of CKD patients included in the study.

Characteristic	Total (n = 1498)	Non-osteoporosis(n = 1245)	Osteoporosis(n = 253)	P-value
Age	63 (51, 74)	61 (48, 72)	72 (63, 80)	< 0.001
Gender				< 0.001
Male	779 (52)	703 (56)	76 (30)	
Female	719 (48)	542 (44)	177 (70)	
Race				< 0.001
Mexican American	233 (16)	195 (16)	38 (15)	
Other Hispanic	123 (8)	102 (8)	21 (8)	
Non-Hispanic White	742 (50)	586 (47)	156 (62)	
Non-Hispanic Black	320 (21)	294 (24)	26 (10)	
Other Race	80 (5)	68 (5)	12 (5)	
Height (cm)	166.4 (159.7, 173.7)	168.2 (161.2, 174.8)	159.6 (154.1, 165.5)	< 0.001
Weight (kg)	78.7 (67, 91.8)	81 (70.4, 94.3)	64.6 (56.8, 74.1)	< 0.001
BMI (kg/m2)	28.1 (24.82, 32.3)	28.75 (25.54, 32.88)	25.4 (22.16, 28.42)	< 0.001
Glucose (mmol/L)	5.5 (4.94, 6.66)	5.5 (4.94, 6.83)	5.38 (4.88, 6.22)	0.048
Total Protein (g/L)	72 (68, 75)	72 (69, 75)	71 (67, 74)	0.007
Albumin, serum (g/L)	42 (39, 44)	42 (39, 44)	42 (39, 43)	0.425
Total Cholesterol (mmol/L)	4.99 (4.29, 5.82)	4.97 (4.32, 5.82)	5.07 (4.27, 5.9)	0.478
HDL (mmol/L)	1.29 (1.06, 1.63)	1.27 (1.03, 1.6)	1.5 (1.16, 1.76)	< 0.001
Alkaline Phosphatase (IU/L)	70 (57, 89)	69 (56, 87)	77 (63, 96)	< 0.001
25OHD2+25OHD3 (nmol/L)	61.6 (43.5, 81.3)	60.4 (42.8, 80.9)	66.3 (47.4, 83.8)	0.022
Potassium (mmol/L)	4 (3.8, 4.3)	4 (3.8, 4.3)	4.1 (3.8, 4.4)	0.014
Sodium (mmol/L)	139 (138, 141)	139 (138, 141)	140 (138, 141)	0.028
Calcium (mmol/L)	2.35 (2.3, 2.42)	2.35 (2.3, 2.42)	2.38 (2.3, 2.42)	0.013
Phosphorus (mmol/L)	1.2 (1.07, 1.32)	1.2 (1.07, 1.32)	1.23 (1.13, 1.36)	< 0.001
Uric acid (umol/L)	350.9 (285.5, 416.4)	350.9 (291.5, 416.4)	327.1 (267.7, 386.6)	< 0.001
Creatinine, serum (umol/L)	90.17 (70.72, 114.92)	90.17 (70.72, 114.92)	88.4 (70.72, 106.08)	0.315
Blood Urea Nitrogen (mmol/L)	5.36 (3.93, 7.14)	5.36 (3.93, 7.14)	6.07 (4.28, 7.85)	0.003
Albumin, urine (mg/L)	39.9 (13.53, 111.4)	45.3 (14.4, 119)	24.9 (10.2, 80.5)	< 0.001
Creatinine, urine (mg/dL)	102.5 (61, 153.75)	105 (65, 156)	86 (48, 134)	< 0.001
ACR (mg/g)	43.76 (15.97, 98.86)	45.67 (17.91, 105.66)	35.7 (11.45, 80.98)	0.004
EGFR (mL/min /1.73m^2^)	68.77 (52.98, 96.3)	72.07 (54.06, 98.7)	58.8 (49.55, 83.44)	< 0.001
Smoking status				0.93
No smoking	689 (46)	573 (46)	116 (46)	
Quit smoking	526 (35)	435 (35)	91 (36)	
Current smoking	283 (19)	237 (19)	46 (18)	
Drinking status				< 0.001
No drinking	499 (33)	381 (31)	118 (47)	
Low drinking	954 (64)	824 (66)	130 (51)	
Heavy drinking	45 (3)	40 (3)	5 (2)	
Hypertension				0.101
No	653 (44)	555 (45)	98 (39)	
Yes	845 (56)	690 (55)	155 (61)	
Diabetes				0.008
No	1137 (76)	928 (75)	209 (83)	
Yes	361 (24)	317 (25)	44 (17)	
History of Glucocorticoid use				0.712
No	1385 (92)	1153 (93)	232 (92)	
Yes	113 (8)	92 (7)	21 (8)	
History of fractures				< 0.001
No	988 (66)	850 (68)	138 (55)	
Yes	510 (34)	395 (32)	115 (45)	

BMI, body mass index; HDL, high-density lipoprotein; ACR, urinary albumin-to-creatinine ratio; EGFR, estimated glomerular filtration rate.

**Table 2:** The comparison of patient characteristics between the training cohort and validation cohort.

Characteristic	Total (n = 1498)	Training set(n = 1048)	Validation set(n = 450)	P-value
Age	63 (51, 74)	63 (50, 73)	63 (52, 76)	0.473
Gender				0.956
Male	779 (52)	544 (52)	235 (52)	
Female	719 (48)	504 (48)	215 (48)	
Race				0.612
Mexican American	233 (16)	173 (17)	60 (13)	
Other Hispanic	123 (8)	83 (8)	40 (9)	
Non-Hispanic White	742 (50)	516 (49)	226 (50)	
Non-Hispanic Black	320 (21)	220 (21)	100 (22)	
Other Race	80 (5)	56 (5)	24 (5)	
Height (cm)	166.4 (159.7, 173.7)	166.7 (160, 174.02)	165.8 (159.1, 172.95)	0.115
Weight (kg)	78.7 (67, 91.8)	79.3 (67, 91.82)	77.2 (67.12, 91.7)	0.616
BMI (kg/m2)	28.1 (24.82, 32.3)	28.12 (24.78, 32.25)	28.03 (25.04, 32.31)	0.713
Glucose (mmol/L)	5.5 (4.94, 6.66)	5.44 (4.94, 6.72)	5.5 (4.94, 6.61)	0.797
Total Protein (g/L)	72 (68, 75)	71 (68, 75)	72 (69, 75)	0.378
Albumin, serum (g/L)	42 (39, 44)	42 (39, 44)	42 (39, 44)	0.791
Total Cholesterol (mmol/L)	4.99 (4.29, 5.82)	5.02 (4.32, 5.87)	4.96 (4.27, 5.74)	0.176
HDL (mmol/L)	1.29 (1.06, 1.63)	1.27 (1.06, 1.6)	1.33 (1.06, 1.66)	0.115
Alkaline Phosphatase (IU/L)	70 (57, 89)	71 (57, 89)	69 (56, 88)	0.222
25OHD2+25OHD3 (nmol/L)	61.6 (43.5, 81.3)	60.7 (43.27, 80.23)	63.1 (44.47, 84.42)	0.209
Potassium (mmol/L)	4 (3.8, 4.3)	4 (3.8, 4.3)	4 (3.8, 4.3)	0.734
Sodium (mmol/L)	139 (138, 141)	139 (138, 141)	140 (138, 141)	0.188
Calcium (mmol/L)	2.35 (2.3, 2.42)	2.35 (2.3, 2.42)	2.35 (2.3, 2.42)	0.28
Phosphorus (mmol/L)	1.2 (1.07, 1.32)	1.2 (1.07, 1.32)	1.2 (1.07, 1.32)	0.69
Uric acid (umol/L)	350.9 (285.5, 416.4)	345 (285.5, 410.4)	350.9 (285.5, 416.4)	0.948
Creatinine, serum (umol/L)	90.17 (70.72, 114.92)	90.17 (70.72, 113.37)	90.17 (72.49, 114.92)	0.397
Blood Urea Nitrogen (mmol/L)	5.36 (3.93, 7.14)	5.36 (3.93, 7.14)	5.36 (4.28, 7.14)	0.523
Albumin, urine (mg/L)	39.9 (13.53, 111.4)	38.05 (13.17, 111.25)	47.6 (14.25, 113.2)	0.344
Creatinine, urine (mg/dL)	102.5 (61, 153.75)	102 (60.75, 153.25)	103.5 (61.25, 153.75)	0.846
ACR (mg/g)	43.76 (15.97, 98.86)	42.59 (15.48, 100)	48.09 (17.73, 95.77)	0.328
EGFR (mL/min /1.73m^2^)	68.77 (52.98, 96.3)	69.24 (53.43, 96.88)	67.25 (51.14, 94.02)	0.269
Smoking status				0.778
No smoking	689 (46)	483 (46)	206 (46)	
Quit smoking	526 (35)	363 (35)	163 (36)	
Current smoking	283 (19)	202 (19)	81 (18)	
Drinking status				0.966
No drinking	499 (33)	351 (33)	148 (33)	
Low drinking	954 (64)	666 (64)	288 (64)	
Heavy drinking	45 (3)	31 (3)	14 (3)	
Hypertension				0.52
No	653 (44)	463 (44)	190 (42)	
Yes	845 (56)	585 (56)	260 (58)	
Diabetes				0.994
No	1137 (76)	796 (76)	341 (76)	
Yes	361 (24)	252 (24)	109 (24)	
History of Glucocorticoid use				0.906
No	1385 (92)	970 (93)	415 (92)	
Yes	113 (8)	78 (7)	35 (8)	
History of fractures				0.933
No	988 (66)	690 (66)	298 (66)	
Yes	510 (34)	358 (34)	152 (34)	

BMI, body mass index; HDL, high-density lipoprotein; ACR, urinary albumin-to-creatinine ratio; EGFR, estimated glomerular filtration rate.

**Fig 2:**
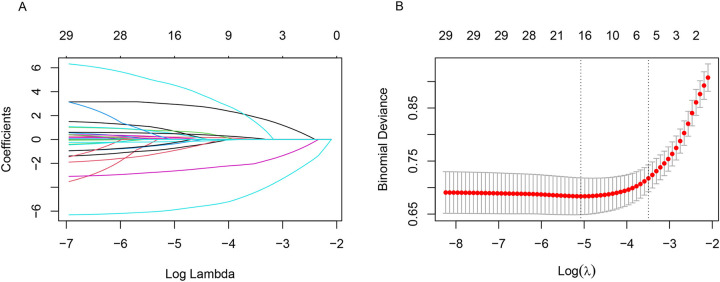
The variation characteristics of the coefficient of variables. The selection process of the optimum value of the parameter lambda in the Lasso regression model. LASSO, least absolute shrinkage and selection operator.

**Fig 3:**
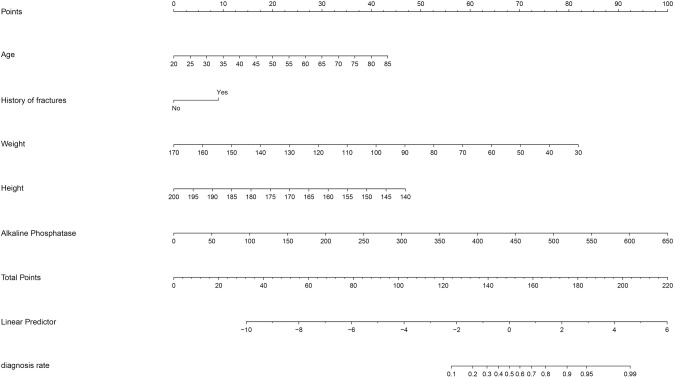
Nomogram prediction model for risk of osteoporosis in CKD patients.

The LASSO regression analysis was conducted on the initial set of 29 potential predictors, and while optimizing the parameters using 1 standard error of the minimum criterion and ten-fold cross-validation (the optimal lambda value was 0.0303), we obtained a subset of 5 optimal variables including age, height, weight, ALP, history of fractures (Fig 2). The multivariate logistic regression analysis revealed significant differences (p < 0.001) in all five optimal variables between individuals with and without osteoporosis. Subsequently, a nomogram was constructed using these five variables to predict the risk of osteoporosis in patients with CKD (Fig 3). In the case of a 60-year-old CKD patient, measuring 170cm in height and weighing 60kg, exhibiting an ALP of 200IU/L, and presenting a history of fracture, the total points amount to 155, indicating a probability of osteoporosis at approximately 65%.

**Fig 4:**
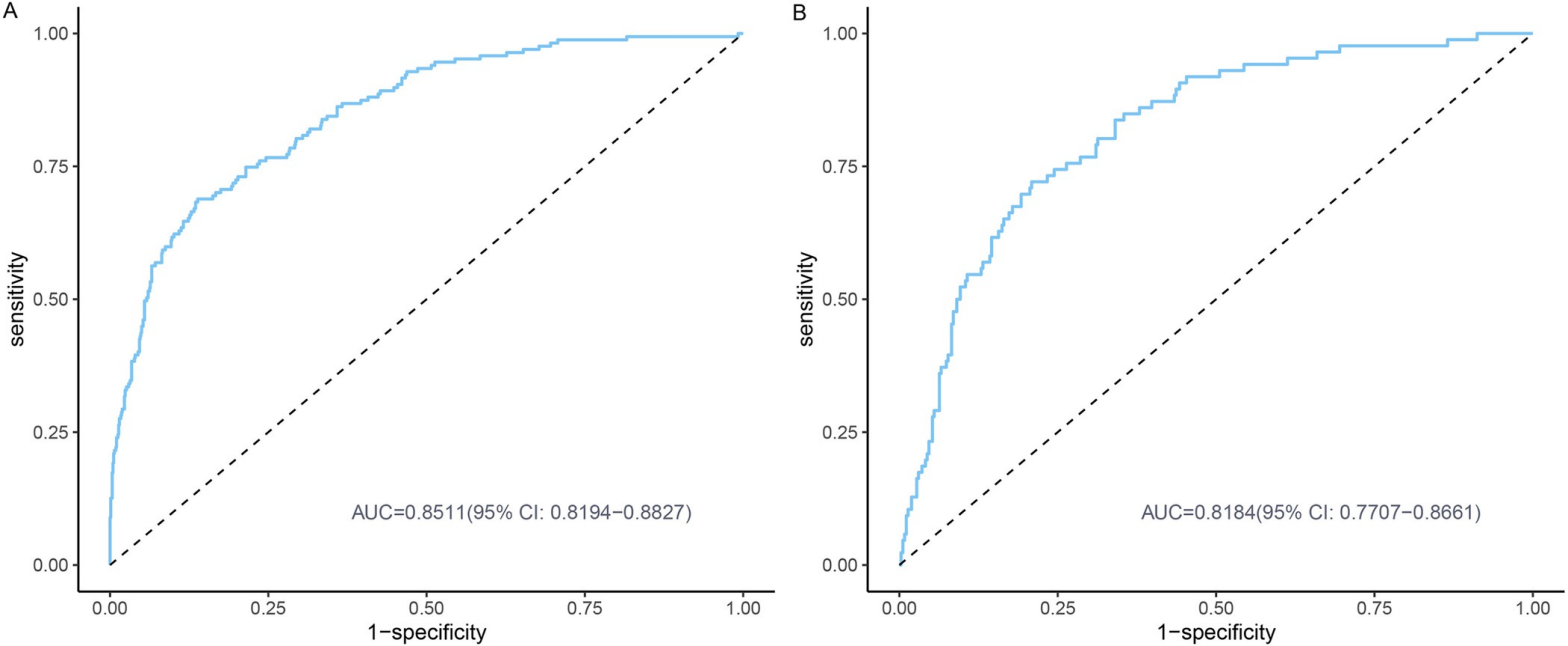
The ROC curve for the training set. The ROC curve for the validation set. ROC, the receiver operating characteristic.

**Fig 5:**
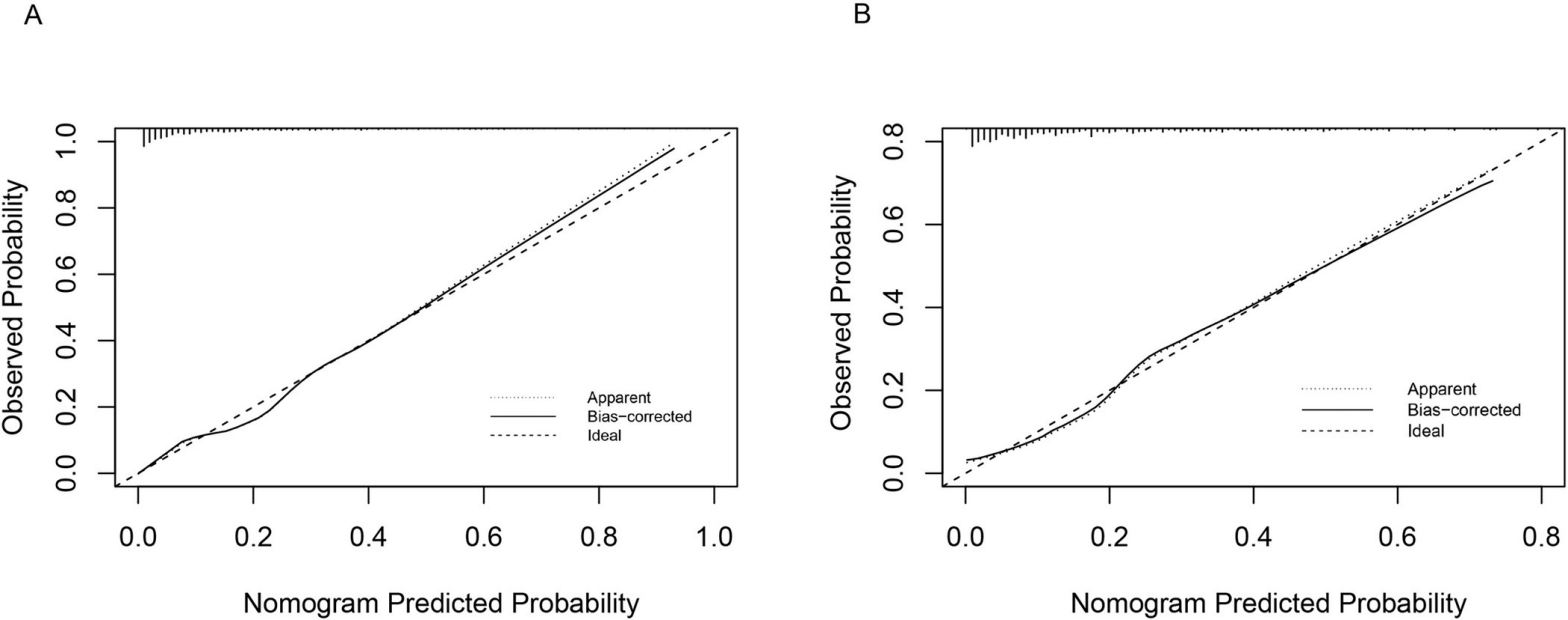
(A) The calibration curve for the training set. (B)The calibration curve for the validation set.

**Fig 6:**
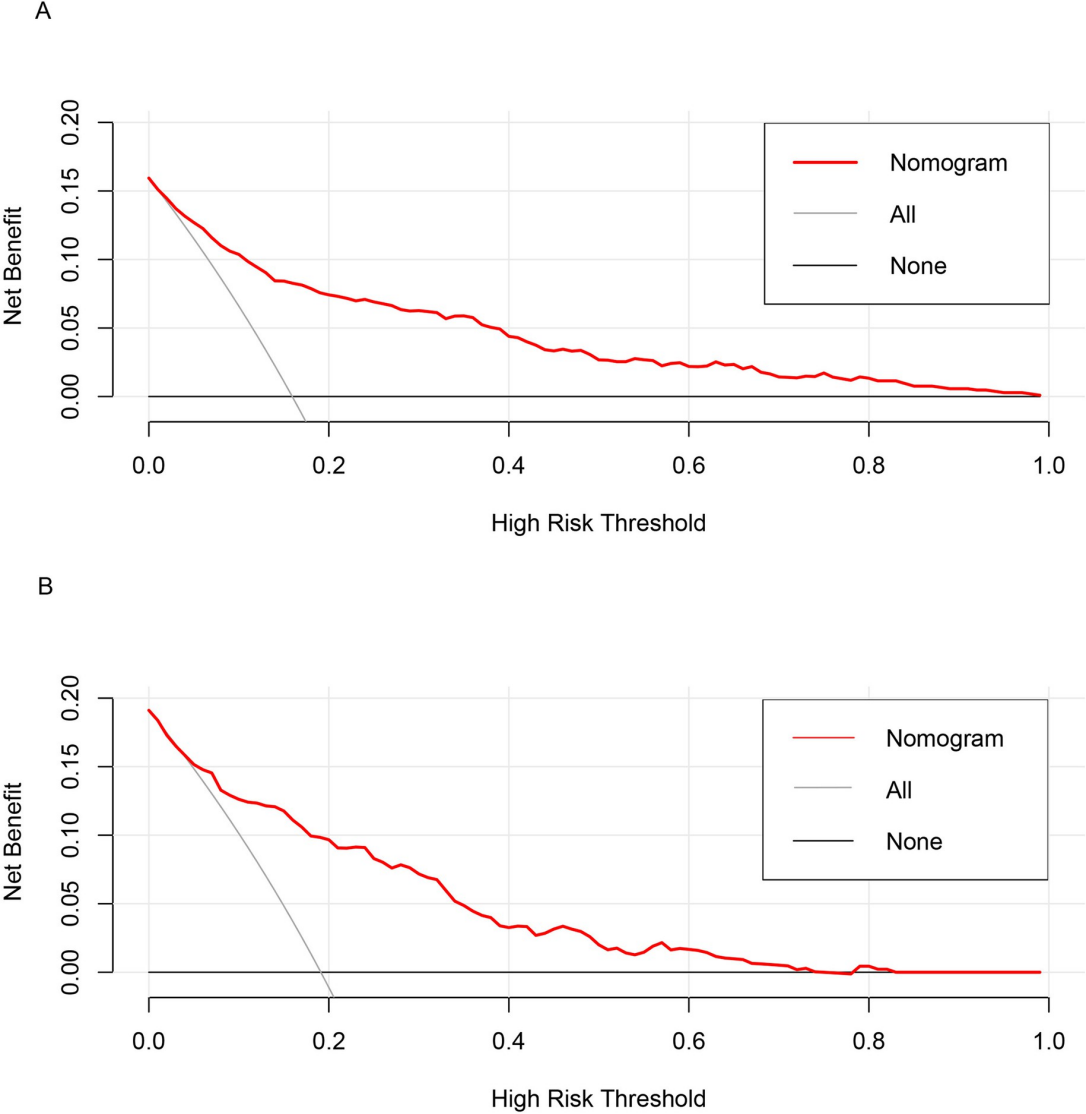
The DCA for the training set. The DCA for the training set. DCA, decision curve analysis.

The AUC for the training model and internal validation were 0.8511 (95%CI 0.8194-0.8827) and 0.8184 (95%CI 0.7707-0.8661), respectively, indicating great discrimination and predictive capabilities of our model (Fig 4). In the calibration curve, the apparent line stands for the probability of direct prediction according to the model. The ideal line stands for a perfect forecast situation. The proximity between these two lines in either the training set or the validation set was well-examined in our study (Fig 5). The Hosmer-Lemeshow test was also conducted on two datasets, yielding P values of 0.475 and 0.156. As evidenced by the results of the aforementioned two evaluation methods, the nomogram demonstrated a high level of calibration. Regarding the clinical applicability of the model, the DCA demonstrated that the net benefit probability ranged from 3% to 98% in the training set and 5% to 70% in the validation set (Fig 6). Specifically, when considering a threshold probability range of osteoporosis in CKD patients between 3% and 98%, utilizing this nomogram yielded superior net benefits compared to either implementing all interventions or no intervention.

## Discussion

In this study, we developed a predictive model using data from 1498 participants obtained from NHANES to predict the risk of osteoporosis in CKD patients. The model incorporated five variables: age, height, weight, ALP, and history of fractures, demonstrating great discriminability, calibration, and clinical applicability in both the training and validation sets.

The model generated in our study showed a positive correlation between age and ALP with the presence of osteoporosis in patients with CKD. The decline in kidney function is common among the elderly population, and a retrospective study conducted on 9931 individuals aged 65 years and above in Ontario, USA revealed an age-associated increase in both elevated serum creatinine levels and decreased GFR [19]. However, it is noteworthy that not all declines in GFR among older adults are pathological processes; normal physiological aging can also cause declines in GFR. This often leads to the overdiagnosis of CKD in the elderly population [20]. Although we employed the age-calibrated CKD-EPI equation, errors may still be present. As for osteoporosis, its progression is also related to age, which has been well-established in previous studies [21]. The ALP is a group of isoenzymes rather than a single enzyme. Within the serum of healthy adults, the most prominent isomers originate from bone and liver sources. Consequently, in individuals with normal hepatic function, an elevation in total ALP levels typically signifies an augmentation in bone-specific ALP activity [22, 23]. Due to its reflection of bone tissue transition, bone ALP can serve as a valuable biomarker for CKD-MBD [24]. In laboratory investigations and clinical observations, the association between ALP and vascular calcification as well as mortality has been observed in patients with CKD-MBD [25, 26]. In comparison to other blood biomarkers, ALP may be a more appropriate choice for osteoporosis screening in patients with CKD. The clinical utility of osteocalcin in renal disease is limited due to its substantial dependence on renal function [27]. The meaning of parathyroid hormones (PTH) depends on its growth trend, rather than solely relying on a single measurement [28].

The risk of developing osteoporosis is inversely correlated to height and weight, suggesting that patients with smaller stature are more susceptible to this condition.

The relationship between weight and CKD is complex. It is currently believed that obese patients may have a higher risk of developing and progressing CKD [29, 30]. However, as renal function further declines and dialysis therapy is implemented, the weight loss persists and becomes significantly associated with patient mortality [31]. This result may be attributed to the fact that CKD is a chronic progressive wasting disease. Additionally, the higher prevalence of osteoporosis among patients with smaller stature may be influenced by gender and age. Older adults and females tend to present smaller height and weight, alongside a high likelihood of osteoporosis [19, 21, 32].

History of fractures was also identified as an independent risk in our study. Fragility fractures, a complication of osteoporosis, also serve as an indicator for assessing the severity of this condition. According to the World Health Organization’s definition of osteoporosis, severe osteoporosis can be diagnosed when a fragility fracture occurs in individuals with existing osteoporosis [17]. Patients with CKD often experience muscle atrophy or cognitive impairment, which increases the likelihood of falls and other adverse events [33, 34]. Those factors may also contribute to an elevated in the incidence of fractures.

Our model contains only five predictors that can be easily obtained through clinical assessment. With the provided medical history and biochemical results, clinicians can accurately calculate the probability of osteoporosis in minutes. For individuals at a high risk of osteoporosis, specialized diagnostic measures such as blood biochemical markers, bone turnover markers, DXA, and even bone needle biopsy can be further conducted to define the diagnosis and specify the treatment. In comparison with prediction models in the general population or patients with high-risk osteoporosis [35–38], the AUC was slightly worse than the articles by Han D et al [36] and Wu S et al [37]. However, the model we developed is more concise than similar studies. Overall, the performance of our model performs quite well while relying on a reduced number of predictors. Table 3 shows more information about these articles.

**Table 3:** The comparison of predictive models between this study and similar research.

Author	Number of patients	Theme	AUC (training set)	AUC (validation set)	Predictors
This study	1498	Predictive model for the risk of osteoporosis in patients with chronic kidney disease	0.851	0.818	Age, Height, Weight, ALP, History of fractures
Wang J et al. [35]	584	Clinical Prediction Model for Predicting Osteoporosis in an Asymptomatic Elderly Population in Beijing	0.761	0.765	Gender, Education level, Weight
Han D et al. [36]	4522	Risk assessment model for osteoporosis-a detailed exploration involving 4552 Shanghai dwellers	0.909	0.903	BMI, TC, TG, HDL, Gender, Age, Education, Income, Sleep, Alcohol Consumption, Diabetes
Wu S et al. [37]	596	Nomogram for predicting osteoporosis in prostate cancer patients: A cross-sectional study from China	0.923	0.859	Age, BMI, Hemoglobin, vitamin D3, testosterone, androgen deprivation therapy duration
Li J et al. [38]	379	Clinical prediction model for osteoporosis in senile patients with type 2 diabetes mellitus	0.844	0.878	Gender, Geriatric nutritional risk index, Serum calcium, Glycated hemoglobin, Duration of diabetes, Serum creatinine

There are some limitations in our study. Firstly, some data were obtained from questionnaires, which may introduce potential bias and compromise the objectivity of our findings. Secondly, certain information related to osteoporosis progression, such as sex hormone levels and PTH, was not accessible or absent from the NHANES database. Lastly, All data used in this study came from a single database, NHANES. It is imperative to employ multi-center data for external validation.

## Conclusion

The study developed and validated a nomogram to predict the risk of osteoporosis in patients with CKD. We anticipate that this nomogram will function as an early warning system, alerting clinicians to conduct timely interventions and referrals for high-risk patients.

## Supporting information

S1 File (CSV)

S2 File (DOCX)
