# Supplementary material for: Construction and validation of a predictive model for the risk of osteoporosis in patients with chronic kidney disease based on NHANES data
Source: PLoS One. 2025 Feb 6;20(2):e0316494. doi: 10.1371/journal.pone.0316494 (PMC11801546; doi:10.1371/journal.pone.0316494)
Supplement: S2 File — (DOCX) [file pone.0316494.s002.docx]

Gender, age, race, history of hypertension and fractures, smoking and drinking status as well as history of glucocorticoid use were obtained through a questionnaire. The full questionnaire is provided in the link below.

**2005-2006:**

<https://wwwn.cdc.gov/nchs/data/nhanes/2005-2006/questionnaires/sp_dmq_d.pdf>

<https://wwwn.cdc.gov/nchs/data/nhanes/2005-2006/questionnaires/sp_smq_d.pdf>

<https://wwwn.cdc.gov/nchs/data/nhanes/2005-2006/questionnaires/mi_alq_d.pdf>

<https://wwwn.cdc.gov/nchs/data/nhanes/2005-2006/questionnaires/sp_bpq_d.pdf>

<https://wwwn.cdc.gov/nchs/data/nhanes/2005-2006/questionnaires/sp_osq_d.pdf>

**2007-2008:**

<https://wwwn.cdc.gov/nchs/data/nhanes/2007-2008/questionnaires/dmq207_08_eng.pdf>

<https://wwwn.cdc.gov/nchs/data/nhanes/2007-2008/questionnaires/smq07_08_eng.pdf>

<https://wwwn.cdc.gov/nchs/data/nhanes/2007-2008/questionnaires/mi_alq_e.pdf>

<https://wwwn.cdc.gov/nchs/data/nhanes/2007-2008/questionnaires/bpq07_08_eng.pdf>

<https://wwwn.cdc.gov/nchs/data/nhanes/2007-2008/questionnaires/osq07_08_eng.pdf>

**2009-2010:**

<https://wwwn.cdc.gov/nchs/data/nhanes/2009-2010/questionnaires/dmq_f.pdf>

<https://wwwn.cdc.gov/nchs/data/nhanes/2009-2010/questionnaires/smq_f.pdf>

<https://wwwn.cdc.gov/nchs/data/nhanes/2009-2010/questionnaires/mi_alq_f.pdf>

<https://wwwn.cdc.gov/nchs/data/nhanes/2009-2010/questionnaires/bpq_f.pdf>

<https://wwwn.cdc.gov/nchs/data/nhanes/2009-2010/questionnaires/osq_f.pdf>

**2013-2014:**

<https://wwwn.cdc.gov/nchs/data/nhanes/2013-2014/questionnaires/DMQ_H.pdf>

<https://wwwn.cdc.gov/nchs/data/nhanes/2013-2014/questionnaires/SMQ_H.pdf>

<https://wwwn.cdc.gov/nchs/data/nhanes/2013-2014/questionnaires/ALQ_CAPI_H.pdf>

<https://wwwn.cdc.gov/nchs/data/nhanes/2013-2014/questionnaires/BPQ_H.pdf>

<https://wwwn.cdc.gov/nchs/data/nhanes/2013-2014/questionnaires/OSQ_H.pdf>

**2017-2018:**

<https://wwwn.cdc.gov/nchs/data/nhanes/2017-2018/questionnaires/DMQ_J.pdf>

<https://wwwn.cdc.gov/nchs/data/nhanes/2017-2018/questionnaires/SMQ_J.pdf>

<https://wwwn.cdc.gov/nchs/data/nhanes/2017-2018/questionnaires/ALQ_CAPI_J.pdf>

<https://wwwn.cdc.gov/nchs/data/nhanes/2017-2018/questionnaires/BPQ_J.pdf>

<https://wwwn.cdc.gov/nchs/data/nhanes/2017-2018/questionnaires/OSQ_J.pdf>
